# Role of osteopontin in bone remodeling and orthodontic tooth movement: a review

**DOI:** 10.1186/s40510-018-0216-2

**Published:** 2018-06-25

**Authors:** Amarjot Singh, Gurveen Gill, Harsimrat Kaur, Mohamed Amhmed, Harpal Jakhu

**Affiliations:** 1 Faculty of Dentistry, McGill University, Montreal, Quebec Canada; 2 Department of Endodontics, Government Dental College, Amritsar, Punjab India; 3 Lady Davis Institute, Jewish General Hospital, Montreal, Quebec Canada; 4 Sandalwood Smiles, Private Dental Practice, Brampton, Ontario Canada

**Keywords:** Osteopontin, Bone remodeling, Biomarkers, Root resorption, Orthodontic tooth movement

## Abstract

In this review, most of the known and postulated mechanisms of osteopontin (OPN) and its role in bone remodeling and orthodontic tooth movement are discussed based on available literature. OPN, a multifunctional protein, is considered crucial for bone remodeling, biomineralization, and periodontal remodeling during mechanical tension and stress (orthodontic tooth movement). It contributes to bone remodeling by promoting osteoclastogenesis and osteoclast activity through CD44- and αvβ3**-**mediated cell signaling. Further, it has a definitive role in bone remodeling by the formation of podosomes, osteoclast survival, and osteoclast motility. OPN has been shown to have a regulatory effect on hydroxyapatite crystal (HAP) growth and potently inhibits the mineralization of osteoblast cultures in a phosphate-dependent manner. Bone remodeling is vital for orthodontic tooth movement. Significant compressive and tensional forces on the periodontium induce the signaling pathways mediated by various osteogenic genes including OPN, bone sialoprotein, Osterix, and osteocalcin. The signaling pathways involved in the regulation of OPN and its effect on the periodontal tissues during orthodontic tooth movement are further discussed in this review. A limited number of studies have suggested the use of OPN as a biomarker to assess orthodontic treatment. Furthermore, the association of single nucleotide polymorphisms (SNPs) in OPN coding gene Spp1 with orthodontically induced root resorption remains largely unexplored. Accordingly, future research directions for OPN are outlined in this review.

## Background

Osteopontin (OPN) is a highly phosphorylated and glycosylated sialoprotein that is expressed by several cell types including osteoblasts, osteocytes, and odontoblasts. OPN belongs to the family of non-collagenous proteins known as SIBLING (*s*mall *i*ntegrin-*b*inding *li*gand, *N*-linked *g*lycoprotein) [1]. In humans, OPN is encoded by Spp1 gene located on the long arm of chromosome 4 region 22 (4q1322.1). OPN is a prominent component of mineralized extracellular matrices of bones and teeth [2]. It has been found to be involved in a number of pathologic and physiological events including bone remodeling, biomineralization, wound healing, apoptosis, and tumor metastasis [2].

Bone remodeling is crucial for maintaining the normal skeletal structure as well as a key factor for orthodontic tooth movement. Orthodontic forces exert a significant amount of compressive [3–9] and tensional [7, 10–13] forces on the periodontium to induce the signaling pathways mediated by various osteogenic genes including OPN, bone sialoprotein, Osterix, and osteocalcin. The signaling pathways and response of the periodontium differ on both tension and compression sides; however, OPN is ubiquitously expressed in bone remodeling on both sides [13].

In this review, our focus will be on the events controlled by OPN in bone remodeling and orthodontic tooth movement. In addition, the prospects of OPN in accelerating tooth movement and root resorption and as a biomarker will be outlined. In our knowledge, no study till date has reviewed the mechanisms involved in OPN-mediated bone remodeling during orthodontic tooth movement.

## OPN structure and its expression and regulation

**Fig. 1:**
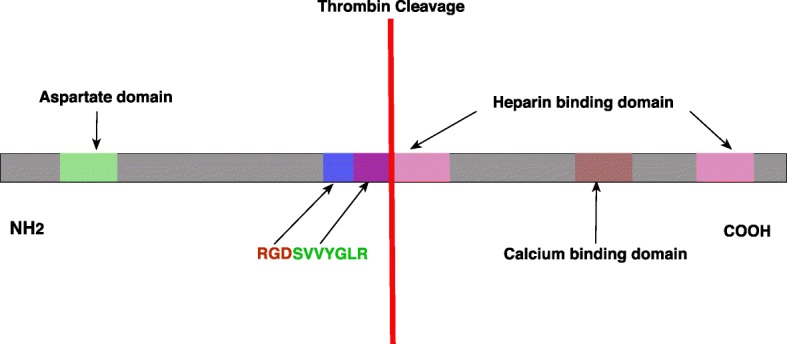
A schematic representation of osteopontin structure and thrombin cleavage site. RGD (arginine-glycine-aspartic acid) and SVVYGLR (serine-valine-valine-tyrosine-glutamate-leucine-arginine) binding domains are indicated

OPN is multifunctional protein owing to its structure. OPN molecule comprises unique conserved regions which involve (RG)-binding domain, serine/threonine phosphorylation site, two heparin-binding sites, one thrombin cleavage site, and a putative calcium-binding site [14]. The cell interacting domains include arginine-glycine-aspartic acid (RGD) cell-binding sequence and serine-valine-valine-tyrosine-glutamate-leucine-arginine (SVVYGLR) motif [15]. The cleavage sites include thrombin and matrix metalloprotinase’s (MMP’s) cleavage sites [14]. In response to cleavage by thrombin, SVVYGLR site is revealed and leads to the formation of two segments: N-terminal fragment and C-terminal fragment (Fig. 1). The pro-inflammatory N-terminal segment includes two integrin-binding sites: RGD and SVVYGLR motifs [15]. However, the C-terminal fragment is devoid of an integrin-binding site. MMP’s cleave both fragments by binding to MMP’s cleavage sites: cleaving N-terminal fragment leads to inactivation of integrin-binding domain of SVVYGLR motifs [15].

The expression of OPN is regulated by a large number of cytokines, hormones, and growth factors, which affects gene transcription, translation, and post-translational modifications (Table 1) [16]. Also, expression of OPN increases in response to mechanical stress [17–19]. Therefore, it is a critical factor in regulating bone remodeling in responses to mechanical stimuli.

**Table 1:** Factors affecting the expression and regulation of osteopontin

Expression and upregulation of OPN	Downregulation of OPN
Transcription factors—Runx2 and Osterix [68]	cGMP-dependent protein kinase [2]
Inorganic phosphate [69]	Bisphosphonates [2]
Systematic conditions—hypophosphatemia, hypocalcemia [2] Hormones—glucocorticoids, [70] 1,25-dihydroxyvitamin D3, [70] parathyroid hormone [14]	ERK inhibitor
Vitamins—retinoic acid [70]	
Inflammatory mediators—TNFα, IL-1β, TGFβ [14]	
Mechanical stress	

## OPN in bone remodeling

**Fig. 2:**
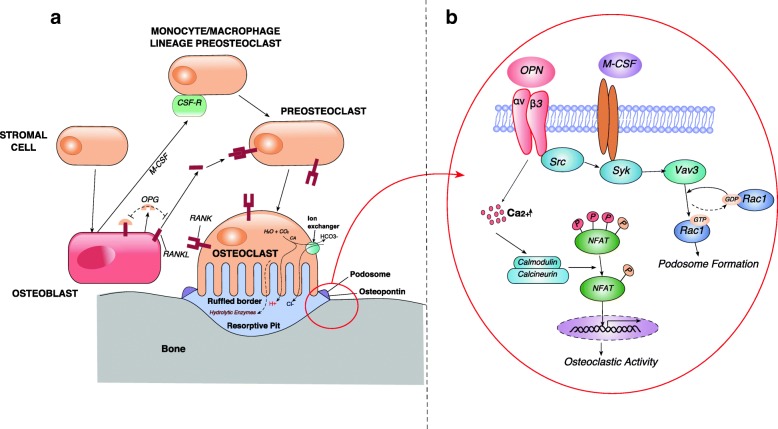
A schematic representation of bone resorption occurring at cellular and molecular level. **a** RANKL/RANK/OPG pathway and osteopontin in podosome formation. **b** Osteopontin binding to integrin αvβ3 leads to podosome formation and osteoclastic activity via Rac and NFAT pathway respectively. M-CSF (macrophage colony-stimulating factor), CSF-R (colony-stimulating factor receptor), RANKL (receptor activator of nuclear kappa-B ligand), RANK (receptor activator of nuclear kappa-B), OPG (osteoprotegerin), Src (proto-oncogene tyrosine-protein kinase), Syk (Spleen tyrosine kinase), Vav3 (vav guanine nucleotide exchange factor 3), Rac1 (Ras-related C3 botulinum toxin substrate 1), NFAT (nuclear factor of activated T cells)

OPN is considered to play important role in bone formation and resorption [20–22]. It is highly concentrated at cement lines where pre-existing and newly formed bone meet and at bone surfaces interfacing with cells called as laminae limitantes [23]. There are various levels of mediation of OPN in bone remodeling. For example, OPN is demonstrated to have chemotactic activity [24] on the precursor of osteoclasts, at a concentration from 10 nM to 1 μM [17]. Also, OPN-dependent intracellular signaling is seen in sealing zone formation in osteoclastic resorption (Fig. 2a, b). Broadly, various authors described the following pathways in OPN-mediated bone remodeling.

### Integrin αvβ3-mediated signaling

OPN binds to several integrins including αvβ3, αvβ5, αvβ1, α4β1, α5, and α9β1. OPN binding to αvβ3 is crucial for major post-receptor signal responses, which involves regulation of osteoclastic activity and activation of osteoprotegerin expression [24, 25]. Further, OPN binding to integrin αvβ3 plays a major role in the formation of sealing zone in osteoclast activity. OPN-αvβ3 binding on the surface of osteoclasts induces integrin clustering and leads to intracellular signaling by phosphorylation of protein tyrosine kinase 2 (PYK2) [25, 26] that facilitate binding of proto-oncogene tyrosine-protein kinase (Src) via its SH2 domain. This Src-PYK2 binding leads to further phosphorylation of PYK2 at other sites which amplifies the signals activating cellular functions including cell adhesion such as sealing zone formation (Fig. 2b) [25, 26].

It has also been suggested that integrin αvβ3, Src, and Fms (the receptor for M-CSF) stimulate Spleen tyrosine kinase (Syk) which further mediates GTP loading on Rac1 via Vav3 in osteoclasts [27]. GTP loading on Rac 1 drives cytoskeletal remodeling leading to bone resorption. Certain proteins including Wiskott-Aldrich syndrome protein (WASP) and gelsolin are also regulated by integrin αvβ3. This process is vital for the podosome formation on osteoclasts [27].

In addition, OPN binding to integrin αvβ3 has been suggested to modulate intracellular Ca^2+^ through stimulation of Ca^2+^ release from intracellular compartments and regulating extracellular calcium influx via Ca^2+^-ATPase pump [28, 29]. The induction of cytosolic Ca^2+^ further modulates osteoclast activity by translocation of transcription factor NFATc1 (nuclear factor of activated T cells, cytoplasmic 1) through the Ca^2+^-NFAT pathway (Fig. 2b) [30, 31]. This NFATc1 has been shown to be imperative for osteoclastogenesis [32–34], leading to the increased resorptive activity of mature osteoclasts [30, 31].

### CD44-associated cell signaling

Osteoclasts deficient in OPN show no migratory activity and do not resorb bone [35]. It has been demonstrated that OPN-deficient osteoclasts, when treated with exogenous OPN, result in an enhanced CD44 expression [36]. CD44-induced cell signaling enhances osteoclast motility [35], which partially restores bone resorption, by activation of αvβ3 integrin [36, 37]. OPN stimulate osteoclast migration through αvβ3- and CD44-mediated cell signaling, which further increases CD44 expression on osteoclasts [35, 36]. Addition of exogenous OPN partially restores the resorptive activity of osteoclasts, which indicates autocrine OPN is important to osteoclast activity [36]. However, exogenously added OPN does not have access to OPN secreted by osteoclasts, which are present in resorption lacuna [36]. The intracellular form of OPN (iOPN), an integral component of the CD44-ERM complex, is seen to be involved in migrating fibroblasts, macrophages, osteoclasts, and metastatic breast cancer lines [2, 38]. A hypothetical pathway was described in which iOPN with components of CD44-ERM is involved in cell migration [2, 38]. Further, it has been demonstrated that overexpression of phosphatase and tensin homolog (PTEN) restricts PI3-kinase signaling, suppresses receptor activator of nuclear kappa-B ligand (RANKL) and OPN-induced Akt activation, and ultimately results in the downregulation of osteoclast differentiation and cell motility [39].

### Inhibition of mineral deposition

The bone matrix consists of the inorganic component, hydroxyapatite (HA), and organic component, proteins and proteoglycans [2]. OPN protein along with other SIBLING proteins contain acidic, serine-, and aspartate-rich motif (ASARM) which are the potential phosphorylation sites [1]. Phosphorylated OPN inhibits mineralization via phosphate residues [40]. Contrary to it, OPN dephosphorylation by tissue-non-specific alkaline phosphatase (TNAP) prevents much of its mineral binding and crystal growth activity [40]. Both pyrophosphate (PPi) and OPN contains highly negative charge phosphate residues which inhibit mineralization after binding to HA crystals [40]. It has been shown that peptide phosphorylated MEPE ASARM (pASARM) has a greater affinity for HA than nonphosphorylated ASARM (npASARM). OPN can act independently of PPi as well as a mediator of PPi effects. High levels of extracellular PPi lead to increased OPN expression and secretion by osteoblasts [40].

Pyrophosphate prevents mineralization by three proposed mechanisms. Firstly, there is direct binding of PPi to growing HA crystals. Secondly, there is the induction of OPN expression by osteoblasts through MAPK pathway, enabling the coordinated action of both PPi and OPN [40]. Thirdly, there is a feedback mechanism in which Pi/PPi ratio inhibits TNAP activity [40]. Even though OPN is considered as mineralization inhibitor, it has been shown that OPN can serve as an agent for intra-fibrillar mineralization in collagen [41], thus pointing towards the multifunctional role of OPN.

## Potential role of OPN in orthodontic tooth movement

Various knockout studies have demonstrated that bone remodeling is impaired in OPN-deficient mice [42] in response to mechanical stress [8, 43, 44]. An animal study [44], by Walker et al., has revealed that OPN is required for osteoclast recruitment through RANKL expression in unloaded mechanical stress (unopposed molar model). Further, it has been suggested that OPN mediates osteoclast activity, RANKL expression, and bone resorption at unloaded alveolar bone walls using a PI3K- and ERK-dependent mechanism [44]. No distal drifting was reported in the OPN-deficient mice [44].

**Fig. 3:**
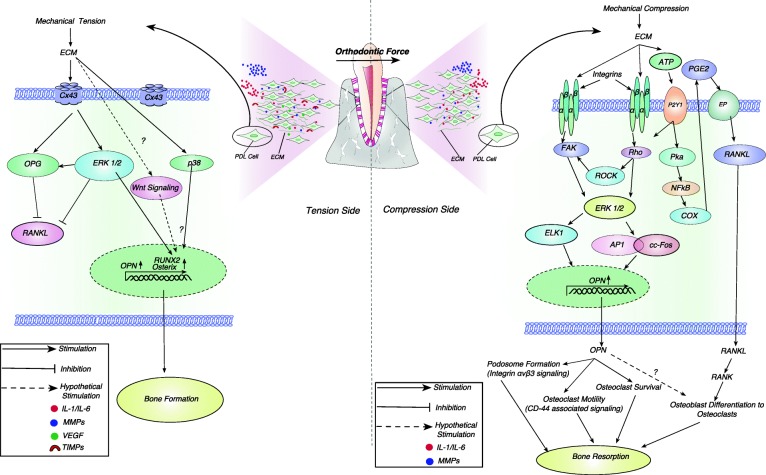
A schematic representation of osteopontin regulation and osteopontin-mediated periodontal remodeling during orthodontic tooth movement at tension side and compression side. ECM (extra-cellular matrix), PDL (periodontal ligament), Cx43 (connexin 43), ERK1/2 (extra-cellular signal-regulated kinase 1,2), RUNX2 (runt-related transcription factor 2), IL-1/IL-8 (interleukin 1/8), MMP’s (matrix metalloproteinases), VEGF (vascular endothelial growth factor), TIMP’s (tissue inhibitors of metalloproteinases), ATP (adenosine triphosphate), PGE2 (prostaglandin E2), EP, RANKL (receptor activator of nuclear kappa-B ligand), RANK (receptor activator of nuclear kappa-B), OPG (osteoprotegerin), P2Y1 (purinoreceptor 1), Pka (protein kinase A), NFkB (nuclear factor kappa B), COX (cyclooxygenase), ROCK (Rho-associated protein kinase), FAK (focal adhesion kinase), ELK1 (ETS domain containing protein), AP1 (activator protein 1)

In the initial stages of orthodontic tooth movement, OPN is observed in the osteocytes [13]. A study [17] suggested the change in the number of OPN mRNA expressing osteocytes on the pressure side after 48 h of mechanical stress and reached a maximum value at 72 h [8], coinciding with bone resorption. However, in the later stages of OTM, OPN is ubiquitously expressed in PDL cells, osteoclasts, cementocytes, cementoblasts, and osteoblasts as well as the cement line of alveolar bone and cementum [13, 45, 46]. The potential signaling pathways involved in the OPN regulation during the orthodontic tooth movement on compression as well as on tension side are summarized in Fig. 3.

### OPN and RANKL regulation on compression side

Wongkhantee and coworkers first studied the OPN expression in human periodontal ligament cell (HPDL) via Rho kinase pathway (Fig. 3) [4] and analyzed that stress-induced ATP activates Rho kinase pathway via the purinoreceptor 1 (P2Y1) receptor [5]. They proposed that RANKL upregulation during mechanical compression may be further induced via activation of NFκB pathway-mediated release of cyclooxygenase and prostaglandin E2 (PGE2) production [3]. Later, various research groups analyzed the Rho kinase-mediated OPN induction. Hong et al. reported that OPN induction during compression is mediated by RhoA-controlled focal adhesion kinase (FAK) and extracellular signal-regulated kinase (ERK) pathways in human periodontal ligament fibroblasts (Fig. 3). ERK further phosphorylates ETS domain-containing protein (Elk-1) which results in the transcription of OPN [47].

OPN and RANKL collectively work to induce the bone resorption in response to compressive forces (Fig. 3). Osteoblasts and stromal stem cells express receptor activator RANKL which binds to its receptor, receptor activator of nuclear kappa-B (RANK), on the surface of osteoclasts and their precursors. This regulates the differentiation of precursors into multinucleated osteoclasts [48, 49]. In addition, a study by Walker and coworkers suggested that increased OPN expression enhances RANKL expression via extracellular matrix signaling pathway in unloaded distal drift [44]. Nevertheless, no study has assessed the influence of OPN expression on RANKL in mechanically stressed condition viz. orthodontic tooth movement and need further investigation.

### OPN regulation on tension side

Su et al. first reported the expression of a gap junction alpha-1 protein, connexin 43, on tension side during orthodontic tooth movement in rat periodontal ligament cells [10]. Later, Shengnan et al. confirmed the involvement of connexin 43 and ERK in tension-induced signal transduction human periodontal ligament fibroblasts (Fig. 3) [7]. It was reported that ERK further induces the transcription of osteogenic proteins, runt-related transcription factor 2 (RUNX2), osteoprotegerin (OPG), and Osterix [7]. In a recent study, the upregulation of OPN along with alkaline phosphatase, collagen I, osteocalcin, and bone sialoprotein was reported via ERK and p38 MAPK-mediated pathway during orthodontic tooth movement in response to tension stress [11]. Thus, both ERK and p38 were proposed to be significantly involved in periodontal remodeling during orthodontic tooth movement [11].

Wnt/β catenin pathway has been shown to be significantly involved in the matrix formation in response to mechanical strain [50–54]. Whether this pathway is involved in the tension forces created during the orthodontic tooth movement is not yet known. Thus, we hypothesize that strain-induced transduction of Wnt/β catenin could be involved in the upregulation of osteogenic proteins including Osterix and OPN (Fig. 3).

## OPN-mediated tooth root resorption and repair

Root resorption is one of the side effects of the orthodontic treatment and is the result of activity of odontoclasts [45]. A mice study showed odontoclast expressing OPN mRNA appeared on the surface of the active root resorption 5 days after orthodontic movement [45]. Similarly, Chung et al. demonstrated that OPN deficiency has much more enhanced effect on the decrease in the odontoclastic activity than osteoclastic activity [43]. They proposed that abundance of inflammatory regulators in the alveolar bone might overwhelm the deficiency of OPN, thereby having little effect on the bone resorption [43]. In contrast to the alveolar bone, cementum and root surface of the tooth is deficient in the inflammatory mediators, thereby enhanced odontoclastic activity may be the one reason in OPN-deficient mice [43]. Thus, OPN is a crucial factor in force-induced root resorption of tooth [43]. Jimenez-Pellegrin et al. demonstrated that OPN plays a key role in both cementum resorption and repair after orthodontic rotation movement [55].

On the other hand, the role of OPN in cementogenesis followed by mechanical injury was also studied in the epithelial cell rests of Malassez (ECRM) [56, 57]. It has been suggested that ECRM express various osteogenic genes including OPG and OPN [56]. Also, immunohistochemical characteristics of ECRM suggested that it may be significantly involved in the secretion of matrix proteins including OPN to further induce cementum repair followed by mechanical injury [57].

Various research groups studied the single nucleotide polymorphisms (SNPs) in the OPN coding gene Spp1 and its effect on the tooth root resorption [58–60]. Iglesias-Linares and coworkers first reported that OPN gene SNPs (rs9138, rs11730582) are involved in the susceptibility of external root resorption in patients undergoing orthodontic treatment [58]. However, in another study, OPN gene SNPs and its effect on external apical root resorption (EARR) were not confirmed in Czech children [60]. However, the association between individual variability in purinoreceptor (P2X7) and EARR was suggested to be an important factor in the etiopathogenesis of EARR [60]. Iglesias-Linares et al. later implicated the Spp1 gene SNPs to assess the orthodontically induced external apical root resorption (OIEARR) in patients with removable appliances versus fixed appliances [59]. No any predisposition to OIEARR was reported with response to fixed and removable appliances [59].

## Future directions

Since OPN is ubiquitously expressed in periodontal remodeling during orthodontic tooth movement, various research groups have implicated OPN as a biomarker to assess the tissue response with respect to orthodontic treatment [61]. The samples were collected from GCF and a protein levels were assessed [61–65]. DNA methylation biomarkers of Spp1 gene and other osteogenic genes may also be helpful to understand the individual variability in response to orthodontic treatment [66]. Thus, a more tailored and personalized approach [66] can be drawn to treat patients with an increased predisposition to OIEARR via targeting the epigenetic mechanisms. Similarly, micro RNAs targeting the osteogenic genes can be assessed.

Alveolar decortication has been shown to induce the rate of tooth movement via the coupled mechanism of bone resorption and formation in early stages of orthodontic tooth movement [67]. The underlying biomarkers (OPN, osteocalcin, bone sialoprotein) demonstrated increased anabolic activity. Whether the orthodontic tooth movement can be accelerated via targeting the underlying signaling pathways warrants further investigation.

## Conclusions

OPN has a definitive role in the formation of podosomes, osteoclast survival, and osteoclast motility. Various OPN-mediated signaling pathways involved in the periodontal remodeling facilitate orthodontic tooth movement. There is a need to pharmacologically target these signaling pathways in order to decrease the side effects of orthodontic treatment including tooth root resorption in patients with an increased predisposition to OIEARR. In addition, the application of OPN biomarkers should be assessed and compared at proteomic, genomic, and epigenomic levels in order to gain a more tailored orthodontic approach. Nonetheless, there is dire need of validated studies to further translate the relevance of OPN in orthodontic treatment.

## Abbreviations

AP1: Activator protein 1
ATP: Adenosine triphosphate
COX: Cyclooxygenase
CSF-R: Colony-stimulating factor receptor
Cx43: Connexin 43
ECM: Extracellular matrix
ELK1: ETS domain containing protein
EP, P2Y1: Purinoreceptor 1
ERK1/2: Extracellular signal-regulated kinase 1,2
FAK: Focal adhesion kinase
IL-1/IL-8: Interleukin 1/8
M-CSF: Macrophage colony-stimulating factor
MMP’s: Matrix metalloproteinases
NFAT: Nuclear factor of activated T cells
NFkB: Nuclear factor kappa B
OPG: Osteoprotegerin
PDL: Periodontal ligament
PGE2: Prostaglandin E2
Pka: Protein kinase A
Rac1: Ras-related C3 botulinum toxin substrate 1
RANK: Receptor activator of nuclear kappa-B
RANKL: Receptor activator of nuclear kappa-B ligand
RGD: Arginine-glycine-aspartic acid
ROCK: Rho-associated protein kinase
RUNX2: Runt related transcription factor 2
Src: Proto-oncogene tyrosine-protein kinase
SVVYGLR: Serine-valine-valine-tyrosine-glutamate-leucine-arginine
Syk: Spleen tyrosine kinase
TIMP’s: Tissue inhibitors of metalloproteinases
Vav3: Vav guanine nucleotide exchange factor 3
VEGF: Vascular endothelial growth factor
